# Shear‐Mode Direct Piezoelectric Response of Ferroelectric Nematic Liquid Crystals

**DOI:** 10.1002/advs.77907

**Published:** 2026-09-27

**Authors:** Péter Salamon, Marcell Tibor Máthé, Hiroya Nishikawa, Fumito Araoka, Antal Jákli

**Affiliations:** ^1^ Institute For Solid State Physics and Optics HUN‐REN Wigner Research Centre for Physics Budapest Hungary; ^2^ RIKEN Center for Emergent Matter Science Saitama Japan; ^3^ Department of Physics Kent State University Kent Ohio USA; ^4^ Materials Sciences Graduate Program and Advanced Materials and Liquid Crystal Institute Kent State University Kent Ohio USA

**Keywords:** ferroelectric nematic liquid crystals, piezoelectricity, rheology, viscoelasticity

## Abstract

Piezoelectricity (linear coupling between mechanical deformation and electric signal) was originally observed only in solid crystals. Recently, it was discovered that liquid ferroelectric nematic liquid crystals are also piezoelectric. However, so far only their converse piezoelectric signals (applied voltage‐induced mechanical deformation) were measured quantitatively. In this work, we have carried out periodic shear‐induced electric current and oscillatory rheology measurements on the two archetypic ferroelectric nematic compounds, RM734 and DIO. From temperature, frequency and strain dependent results of the first and second harmonic current signals together with the results from oscillatory rheometry, we were able to quantitatively determine the shear‐mode direct piezoelectric coupling constants. These values are similar for both materials and are compared to results of previous converse piezoelectric measurements. We propose a physical mechanism in which the flow alignment of ferroelectric polarization leads to the direct piezoelectric response.

## Graphical abstract

Periodic shear‐induced electric current measurements are reported on two archetypic ferroelectric nematic liquid crystal compounds. From the temperature, frequency and shear angle dependent results of the first and second harmonic current signals together with temperature and oscillatory rheology measurements, the effective piezoelectric coupling constants were determined.

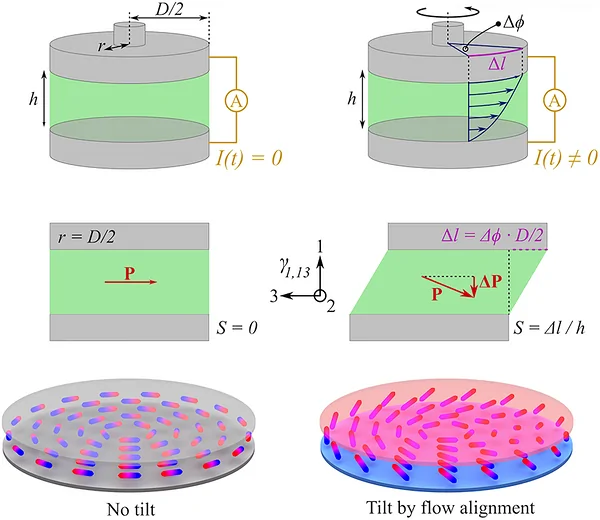

## 1 Introduction

Ferroelectric materials are polar, therefore lack inversion symmetry. As such they are also piezoelectric, i.e., possess linear coupling between mechanical and electric fields (linear electromechanical effects). Ferroelectricity was originally known only in solid crystals, but later it was found in smectic and columnar liquid crystals with one‐ and 2D spatial periodicity [1] and recently, even in nematic liquid crystals with no spatial periodicity but only 3‐D fluid order [2, 3, 4, 5, 6, 7, 8]. In spite of their genuine 3D fluidity, in electric fields they often behave as solid particles or microrobots [9], they can also form metastable freestanding filaments [10, 11] that can be stabilized in small electric fields [11].

Similar to ferroelectricity, piezoelectricity was first observed only in solid crystals, then in polymers. From the late 70s linear electromechanical effects were also observed in ferroelectric smectic phases of chiral rod‐shaped [12, 13] and achiral bent‐core molecules [14], furthermore in the columnar phases of chiral disc‐shaped molecules [15]. Very recently, soon after their discovery in 2017, electromechanical effects were also reported in ferroelectric nematic liquid crystals by Máthé et al. [16]. and Rupnik et al. [17].

The mathematical definition of the direct piezoelectricity, $\Delta P_{i}=\sum_{jk}\gamma_{ijk}T_{jk}$, dictates that the elements of the stress tensor *T_jk_* that induce a polarization component Δ*P_i_* proportional to the elements γ_*ijk*_ of the third rank piezoelectric charge coupling tensor, have to be effective on fluids that flow under DC stress. Similarly, the definition of the converse piezoelectricity, $S_{jk}=\sum_{i}\gamma_{ijk}E_{i}$, requires that the strain *S_jk_* induced by an external electric field applied along *i*, should be small and reversible. Both requirements mean that in fluids we can speak about piezoelectricity only when either the stress in direct effect or the electric field in the converse effect are periodic and small enough to lead to small strain $\hat{S}=\vec{\nabla }\;\cdot \vec{s}$. Here the displacement of a volume element is $\vec{s}\left(t\right)={\vec{s}}_{o}\;\cdot e^{i\omega t}\ll L$, where ω is the angular frequency of the vibration, and *L* is film thickness [18].

**FIGURE 1:**
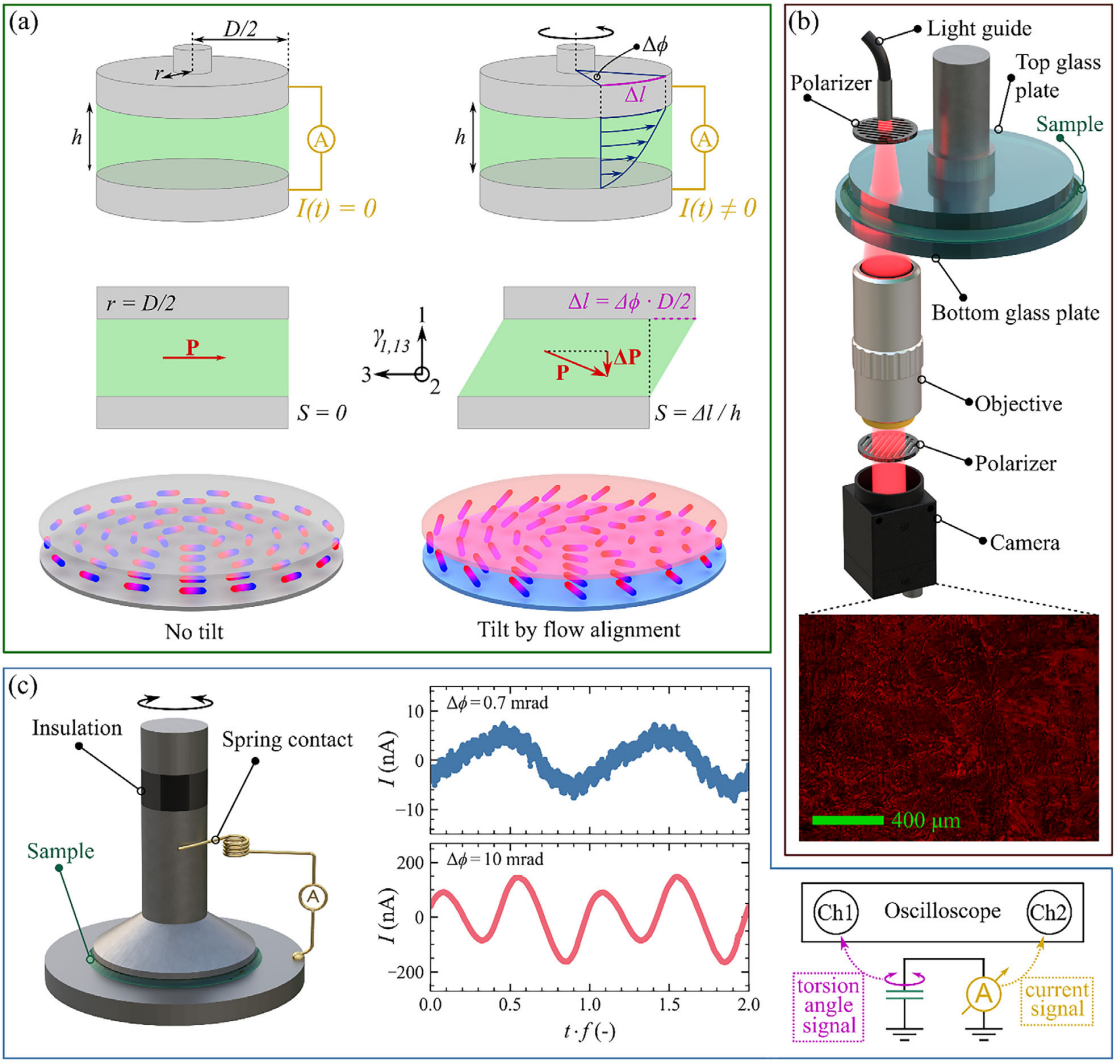
Illustrations of the experimental setup, typical measured current, illustrative micrograph of the texture and the schematic electrical circuit. (a) Sketches of the relevant parameters in the shearing geometry and the proposed shear alignment leading to ***γ***_1,13_. (b) Illustration of the modified rheometer allowing polarizing optical observations in transmission mode, and a typical micrograph of the sample. (c) Illustration of the torsional rheometer for measuring phase dependences of current signals. Schematic electrical circuit of the experimental apparatus. The graphs in the inset show current signals from DIO at 68°C and f=10 Hz at low strain (Δϕ=0.7 mrad) with mainly first harmonic response and with dominant second harmonic at high strain (Δϕ=10 mrad).

So far, quantitatively only some of the converse piezoelectric responses were measured. It was found that in room temperature ferroelectric nematic liquid crystal films aligned parallel to the substrates along axis 3, an electric field applied between the substrates (along 1 in Figure 1), there is a periodic vibration along the polarization corresponding to $\gamma_{1,13}∼3\;\frac{\mathrm{nm}}{V}=3\;\mathrm{nC}/N$ below f∼1kHz and at higher frequencies the piezoelectric coupling constant is inversely proportional to the frequency [18]. These values are larger than that of the strongest piezoelectric crystals and comparable to that of soft ferroelectrets [19, 20, 21, 22] due to the softness of the ferroelectric nematic fluids with loss moduli $G^{\prime \prime }=\eta \cdot \omega$, where η∼0.1−1Pa·s is a typical flow viscosity of room temperature FNLC materials. This angular frequency dependence of the loss modulus explains the observed γ_1,13_ ∝ ω^−1^ for the N_F_ fluids [18]. Interestingly, converse piezoelectric response was also measured normal to the substrates (direction 3 in Figure 1) along the applied electric field with frequency dependences consisting of a set of resonant peaks in the kHz frequency range [16, 18]. This response is not expected by symmetry and likely related to imperfect alignment, a pretilt or the fact that the top plate is not exactly parallel to the bottom one, therefore a motion in 1 direction is coupled to a vertical motion (direction 3).

Direct piezoelectric‐type response was observed by Rupnik et al. [17]. using a simple demonstrator device, where the ferroelectric nematic liquid was placed into a deformable container with electrodes, and the electric current induced by both periodic and irregular actuation of the container was examined. They found that 100 Hz actuation frequency and 100 kΩ load resistance may generate $∼10\;\mu W/\;{\mathrm{cm}}^{2}$ power per unit area which is only 0.01% of the theoretically estimated current and of the specific power produced by solar photovoltaic panels at natural illumination conditions.

In this paper, we use low frequency shear stress to measure direct piezoelectric signals of the two prototypical ferroelectric nematic liquid crystal materials RM734 and DIO and obtain the first quantitative values of the shear piezoelectric charge coupling constant. The values obtained are two orders of magnitude higher than the ones obtained from converse piezoelectric measurements of other ferroelectric nematic liquid crystals. With the help of simultaneous optical observations, we propose a physical model and estimate the maximum shear‐induced electric current one may achieve in uniformly poled samples. Furthermore, we present oscillatory rheological properties of RM734 and DIO, where we found soft elastic behavior in the antiferroelectric intermediate phase.

## 2 Materials and Methods

In our experiments we used the two archetypic ferroelectric nematic compounds: 2,3′,4′,5′‐tetrafluoro[1,1′‐biphenyl]‐4‐yl 2,6‐difluoro‐4‐(5‐propyl‐1,3‐dioxan‐2‐yl) benzoate (DIO) [3], and 4‐[(4‐nitrophenoxy)carbonyl] phenyl‐2,4‐dimethoxybenzoate (RM734 [2] from Instec, USA). The reported phase sequences on cooling of DIO and RM734 are I 158°C N 84.5°C SmZ_A_ 68.8°C N_F_ and Iso – 189°C −N – 133°C −N_F_ ∼89°C, respectively. Here SmZ_A_ is an antiferroelectric phase [23], also called as splay nematic [24] (N_s_).

All measurements were carried out using an Anton Paar MCR502 torsional rheometer in oscillatory mode (Figure 1). The lower plate was fixed, the upper plate was oscillated with the torsion angle ϕ(t)=Δϕ·sin(2πft), where Δϕ is the amplitude of the angle oscillation, *f* is the frequency and *t* is the time. The rheometer is operated in parallel plate geometry with gap *h* = 80 µm between plates and diameter *D* = 25 mm of the disc‐shape plates. The strain *S* at the circumference is $S_{D/2}=\frac{D}{2}\;\frac{\Delta \phi }{h}$. This means that Δϕ=1mrad, corresponds to S_*D*/2_ = 0.15625 = 15.625%. Sketches of the relevant parameters in the shear geometry and the proposed shear alignment leading to γ_1,13_ are shown in Figure 1a.

For optical observations, we performed experiments with RM734 in the ferroelectric nematic phase at 125°C in between transparent indium‐tin‐oxide (ITO) coated parallel plates during oscillatory shear using the rheo‐optical module of the rheometer with custom modifications (see Figure 1b). The diameter of upper rotating plate was 40 mm, and the middle of the field of view was at *r* = 17.7 mm radius and, similar to all other experiments, the gap was *h* = 80 µm. The periodically sheared sample between crossed polarizers was illuminated with a filtered monochromatic LED light source at λ = 660 nm wavelength. The vertical axis of the images was placed at ± 45° with respect to the polarizers and approximately tangential to the direction of motion of the upper plate. Images were taken by a FLIR BFS‐U3‐28S5M‐C camera as a function of phase α = 2 π*ft* of the strain. Note that images (see an example in the inset of Figure 1b) corresponding to increasing phases were taken in subsequent periods at increasing delays of the camera trigger with respect to the actual strain.

The torsional rheometer modified for measuring current signals is illustrated in Figure 1c. The graphs of the phase dependence of the current generated in DIO at 68°C and *f* = 10 Hz at low strain (Δϕ=0.7 mrad) with mainly first harmonic response and with dominant second harmonic at high strain (Δϕ=10 mrad), are also shown in Figure 1c.

A single filling of the rheometer with *D* = 25 mm diameter measuring system required about 120 *mg* of material, which was used almost for a week. The samples were kept in the rheometer under 180 *L*/*h* continuously heated N_2_ stream to avoid degradation and maintain the desired temperature. Except for the optical measurements, the rheometer was equipped with the dielectro‐rheological module and a PP25/DI/TI measuring system allowing floating electrical connections to the bottom and top sides of the sheared fluid. The electric connection at the moving upper plate was achieved by a gold sliding spring contact (see Figure 1b).

The shear induced electrical current between the lower and upper plates was measured by a low‐noise current preamplifier (SR570, Stanford Research Systems, USA). The same *c_I_* =  50 nA/V current conversion factor was used for all measurements. The time dependence of the oscillation angle and the induced current were monitored simultaneously in channel 1 and channel 2 of an oscilloscope (TiePie Handyscope HS5, The Netherlands). The first and second harmonic signals were calculated by digital signal processing using lock‐in technique, as *I*_1*f*_ (*t*) = *I*_1*f*_  · sin (2π*ft* + ψ_1*f*_) and *I*_2*f*_ (*t*) = *I*_2*f*_  · sin (4π*ft* + ψ_2*f*_), where *I*_1*f*_ and *I*_2*f*_ are the amplitudes and ψ_1*f*_ and ψ_2*f*_ are the phase of the induced current, respectively. Typical unprocessed current signals of DIO at 68°C and *f* = 10 Hz can be seen in Figure 1c.

## 3 Results

**FIGURE 2:**
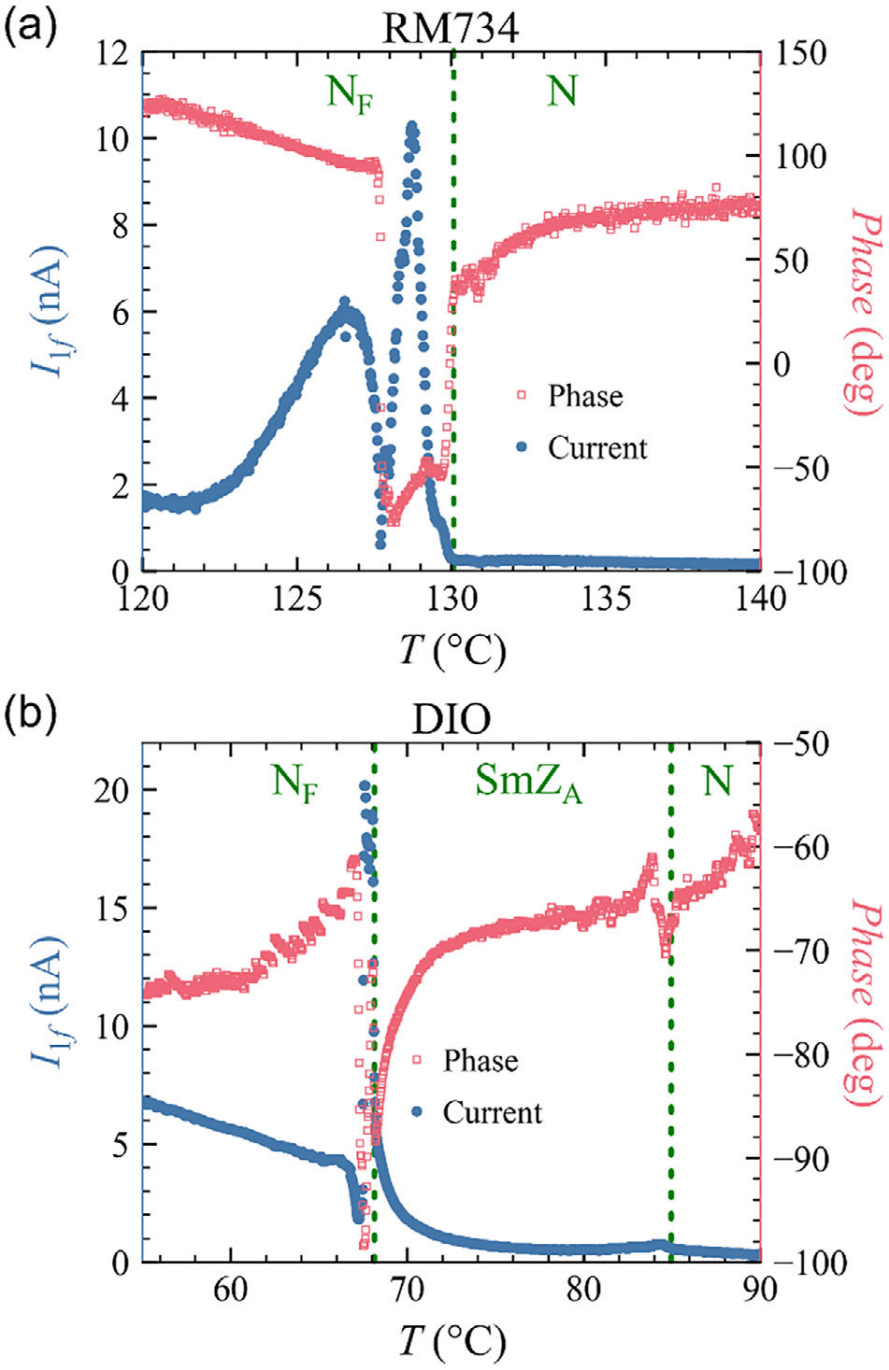
Temperature dependences of the ***I***_1 ***f***_ signals (amplitude and phase) induced by ***f*** = 10 *** Hz***, Δϕ=1mrad periodic shear for RM734 (a) and DIO (b).

Temperature dependences of periodic shear induced currents and the phases for RM734 and DIO measured under 0.5°C/min cooling rate at frequency of *f* = 10 Hz and oscillation amplitude of Δϕ=1mrad are shown in Figure 2a,b, respectively.

In the nematic (N) phase of both materials the induced current is very small (< 0.2 nA). In spite of it, the fluctuation of the phase that decreases at < 5 ^○^/^○^ *C* rate is small indicating that the signal is greater than the measurement error. The induced current increases considerably upon transition to the N_F_ phase of RM734 and to the SmZ_A_ and N_F_ of DIO. For both materials, the temperature dependences of the current have maxima in 1°C–2°C range below the phase transitions. Specifically, for RM734 the current rises to about 10 nA at ≈ 1.5 ^○^C below the N‐N_F_ transition, then decreases to about 0.6 nA by 2.2°C below the transition. Simultaneously the phase drops by about 90° indicating a considerable change in the dynamical response of the material. On cooling below this range, the phase increases by ∼180°. Simultaneously, the current increases up to about ≈ 6 nA at ≈ 127°C, then slowly decreases to about ≈ 1.7 nA by 120 ^○^ *C*. We note that the 2.2°C temperature range below the N phase is close to the range where recent calorimetric studies showed the presence of an intermediate phase [25] which was shown to depend on the ionic content and is assigned to either an antiferroelectric smectic Z (SmZ_A_) phase [26] or to a double splayed nematic phase [27].

In DIO, the peak currents reach 0.8 nA, and 20 nA upon the N‐SmZ_A_ and SmZ_A_‐ N_F_ transitions, respectively. Simultaneously, in both transitions there is a transient decrease of the phase. In contrast to RM734, on cooling further in the N_F_ phase, the amplitude of the shear‐induced piezo current increases.

**FIGURE 3:**
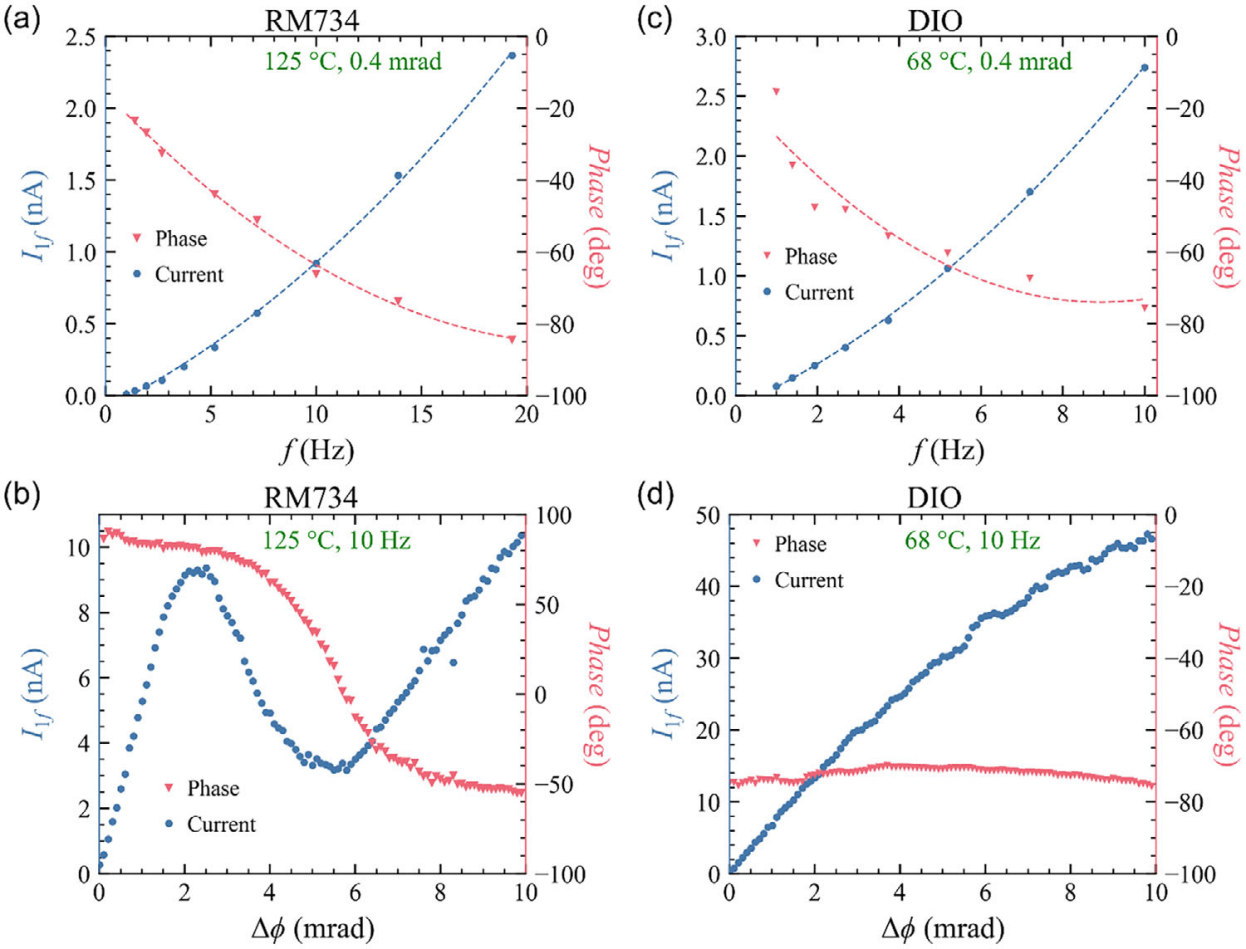
Frequency and the oscillation angle dependences of the induced first harmonic current in the N_F_ phase in RM734 at 125°C and in DIO at 68°C. (a) and (c): Frequency dependences at Δϕ=0.4mrad; (b) and (d): Δϕ dependences at ***f*** = 10 *** Hz***.

The frequency (*f*) and the oscillation angle (Δϕ) dependences of the first harmonic current amplitude (*I*_1*f*_) in RM734 in the N_F_ phase at 125 ^○^ *C* are shown in Figure 3a,b, respectively. For DIO at 68°C in the N_F_ phase the corresponding graphs are presented in Figure 3c,d. For both materials, the frequency dependences of current can be well fitted by second order polynomials (dashed lines in Figure 3a,c) with more than an order of magnitude higher linear than quadratic coefficients. In RM734 (Figure 3a), the phase decreases smoothly by about 90° which may indicate variation of the mechanical response likely due to flow alignment. In DIO (Figure 3c), the frequency dependence is similar to that of RM734. For RM734 (Figure 3b) at *f* = 10 Hz up to Δϕ≈2mrad (corresponding to ${\dot{S}}_{D/2}\leq 4\;s^{-1}$ shear‐rate) $I_{1f}\propto \;\Delta \phi$. In the 2mrad≤Δϕ≤6mrad range the induced current is decreasing and the phase shifts by about 90°. For Δϕ≥6mrad the current increases linearly again but the phase makes about 180° with respect to the low shear rate values, indicating opposite direction of the motion. In DIO, *I*_1*f*_ vs Δϕ is monotonous in the entire measured Δϕ range with linear behavior up to Δϕ≈ 4 mrad.

**FIGURE 4:**
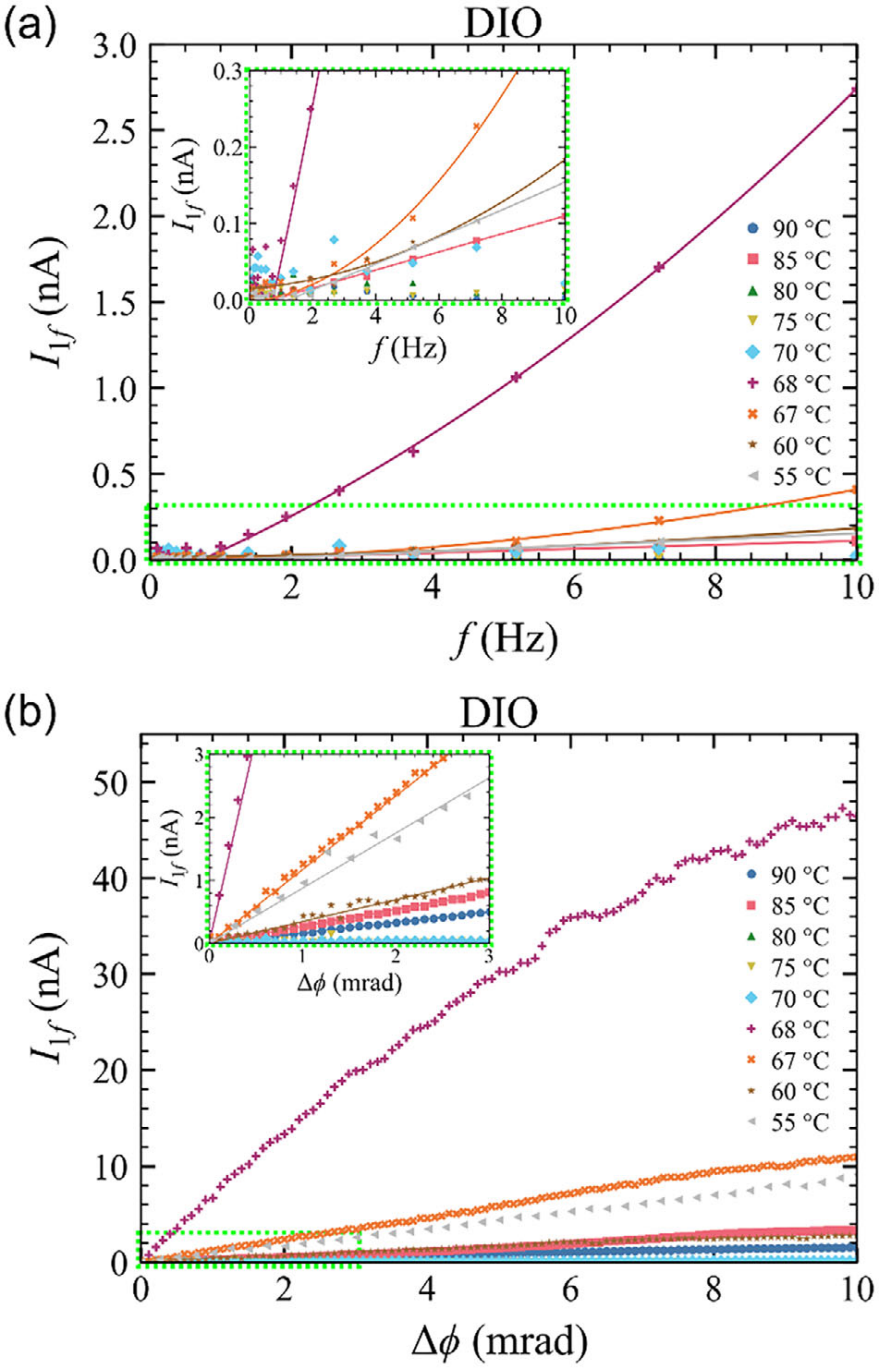
Frequency and oscillation angle dependences of the induced current in DIO at various temperatures between 55°C and 90°C. (a) Frequency dependences at Δϕ=0.4mrad; (b) Δϕ dependences at ***f*** = 10 *** Hz***.

The *f* and Δϕ dependences of the induced current in DIO are shown at various temperatures between 55°C and 90°C (in the N_F_, SmZ_A_ and N phases) in Figure 4a,b, respectively. In accordance with the temperature dependence seen in Figure 2b, the highest signal is measured at 68°C upon the SmZ_A_‐N_F_ transition, and in the vertical axis scale (up to 3 nA) the signals at all other temperatures look very small (within the range marked by the green dotted rectangle). For this reason, the range of the green dotted rectangle (up to 0.3 nA) is blown up in the inset showing that, except in the N phase, the current is proportional to the frequency, i.e., to the shear rate. We see that the data is well fitted by second order polynomials (solid lines in Figure 4a).

Similarly to the frequency dependence, the Δϕ dependence is also the largest at 68°C with a linear increase (Figure 4b). The inset shows the response up to Δϕ=3mrad and up to 3 nA range. At all temperatures the currents are proportional to the oscillation angle up to about Δϕ=3mrad, although the slopes are very small in the *N* phase

**FIGURE 5:**
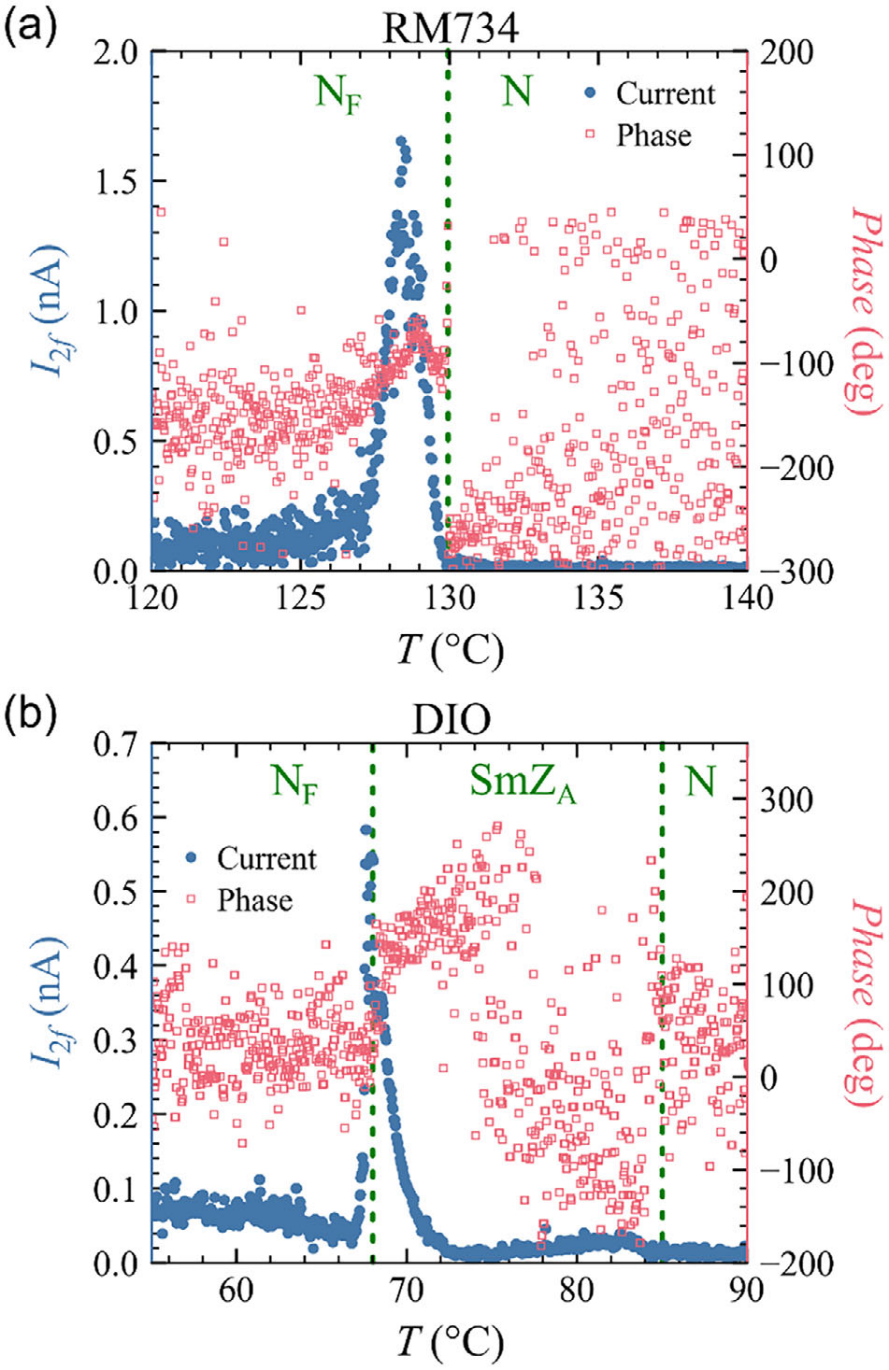
Temperature dependences of the ***I***_2 ***f***_ signals and phases induced by ***f*** = 10 *** Hz***, Δϕ=1mrad periodic shear for RM734 (a) and DIO (b).

The temperature dependences of the second harmonic amplitude *I*_2*f*_ induced by *f* = 10 Hz, Δϕ=1mrad periodic shear are shown for RM734 and DIO in Figure 5a,b, respectively. They were measured simultaneously at the same conditions as of the first harmonic amplitude *I*_1*f*_ seen in Figure 2 and show about an order of magnitude smaller signals than of *I*_1*f*_. Due to this small signal, the phase data are very noisy, especially in the N phase. Just like in the case of the first harmonic signals, the largest responses are measured at the transition to the N_F_ phase. Additionally, in case of DIO, there is a smaller maximum at the N‐SmZ_A_ phase transition.

**FIGURE 6:**
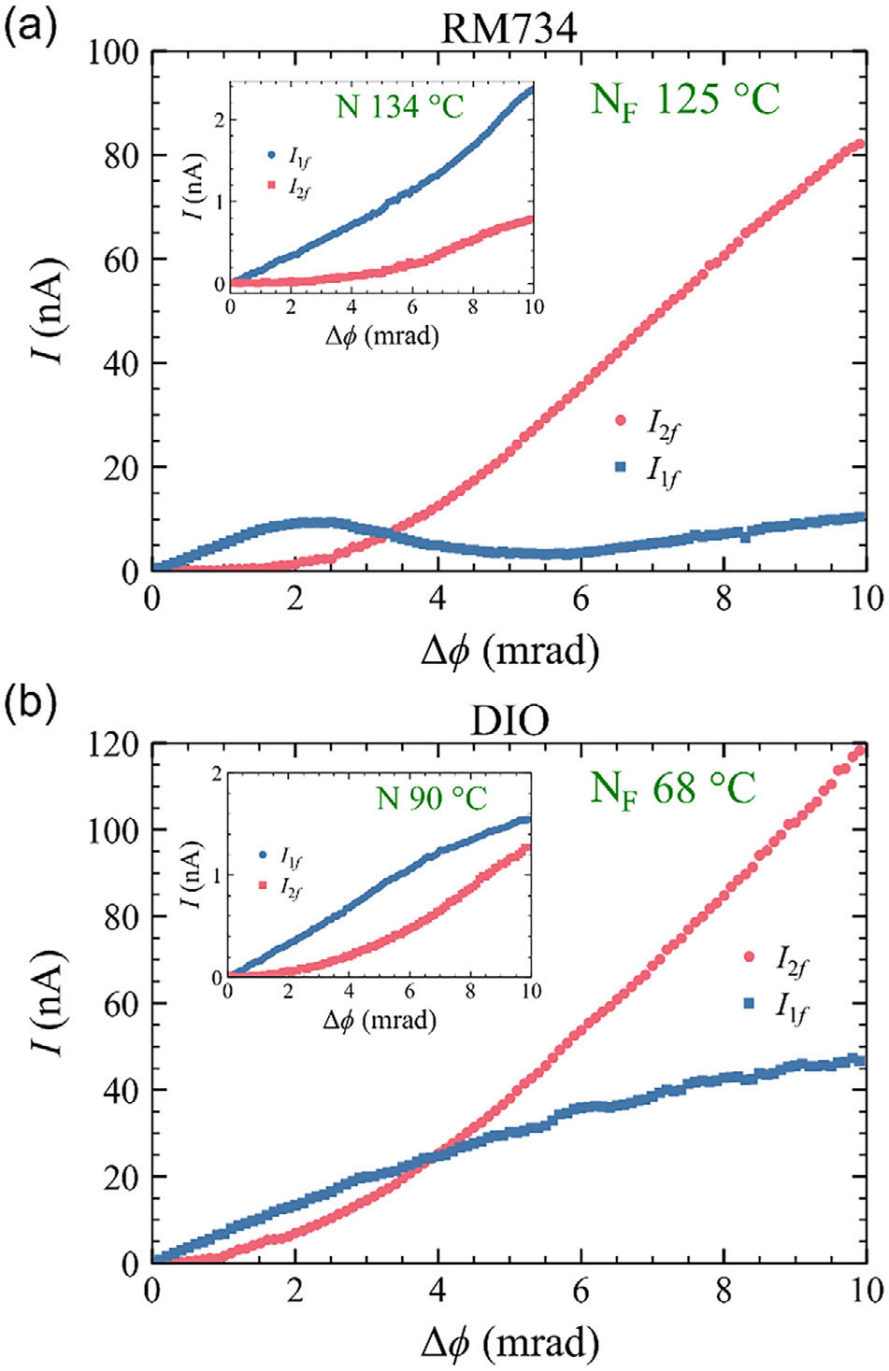
Oscillation angle dependences of ***I***_1 ***f***_ and ***I***_2 ***f***_ at 10 *** Hz***. (a) RM734 at 125°C in the N_F_ phase (main pane) and at 134°C in the N phase (inset); (b) DIO at 68°C in the N_F_ phase (main pane) and at 90°C in the N phase (inset).

From the Δϕ dependences of *I*_1*f*_ and *I*_2*f*_ of RM734 and DIO at 10 Hz shown in Figure 6, we see that the second harmonic signals overcome the first harmonic signals at oscillation angles Δϕ>4mrad.

One can also observe that in the N_F_ phase at f=10HzΔϕ>4mrad ($\dot{S}>4\;s^{-1}$) the slope of *I*_1*f*_ response decreases and the initially quadratic Δϕ dependence of *I*_2*f*_ becomes almost linear. The Δϕ dependence of *I*_1*f*_ and *I*_2*f*_ seen in Figure 6 is likely related to the emergence of the non‐linear response. Small amplitudes of the periodic mechanical excitation only slightly perturb the initial polarization field; therefore, the response signal is linear and free of higher harmonics. At higher amplitudes the deformation is not small anymore. Consequently, the linearity breaks down and a second harmonic component of the response emerges.

In the N phase, both the *I*_1*f*_ and *I*_2*f*_ responses are about 2 orders of magnitudes smaller. We think that the small first harmonic current signal in the nematic phase is a sign of a detectable polar nature. This might be related to a pretransitional phenomenon involving the presence of polar nano clusters even in the N phase [4, 24, 28, 29] but that should strongly decrease on heating. Another possibility is the surface polarization that was detected in several conventional nematic materials [30, 31].

## 4 Discussion

As discussed in a recent review [18], the *N_F_* phase has $C_{\infty \upsilon }$ symmetry, i.e., continuous rotational symmetry about the polar axis, and mirror symmetry through any plane containing the polar axis 3. This means that any 180° rotation around the polar axis 3 (a symmetry operation) changes the sign of 2 (or 1), therefore, components of γ_*i*,*jk*_ containing an odd number of either 1 or 2, must be zero. Thus, the non‐vanishing piezoelectric tensor elements are γ_3,33_, γ_3,11_(  = γ_3,22_ ) and γ_1,13_(  = γ_2,23_  = γ_1,31_  = γ_2,32_ ). In our experiments the polarization direction (along axis 3) can be assumed to be tangential [32, 33, 34, 35, 36], i.e., along the periodic displacement that leads to a velocity gradient normal to substrates (along axis 1), in the direction where we measure the induced current. Therefore, in our studies we measure the piezoelectric charge constant γ_1,31_ = γ_1,13_ . Note that the tangential polarization structure of a cylindrically confined *N_F_* liquid crystal [32, 33, 34, 35, 36] is enforced by the depolarization field that for non‐planar alignment would cost large electrostatic energy (see Figure 1a). In our experiments with metal measuring plate, the tangential director structure is additionally promoted by the concentric circular grooves on the machined surface.

Our experimental results on the induced current *I*_1*f* _ and the measured viscosity data allow us to determine the relevant piezoelectric coupling constant γ_1,13_ using the mathematical definition that Δ *P*_1_ = γ_1,13_  ·  *T*_13_ = γ_1,13_  · *S*_13_ · *G*, where *T*_13_ and *S*_13_ are the relevant stress and strain tensor components, respectively, and *G* is the shear modulus. As an estimate, we identify Δ *P*_1_ = 〈Δ*P*〉  and introduce the average strain $\left\langle S\right\rangle =\frac{2}{3}\;S_{D/2}=\frac{D}{3}\;\frac{\Delta \phi }{h}$ commonly used in parallel plate torsional rheometry [37]. Then the first harmonic current amplitude is approximated by $I_{1f}=fD^{2}\pi^{2}\;\left\langle \Delta P\right\rangle =fD^{2}\pi^{2}\left\langle S\right\rangle \;\gamma_{1,13}G=\frac{fD^{3}\pi^{2}\Delta \phi \left|G^{*}\right|}{3h}\;\gamma_{1,13}$. Using the complex viscosity $|\eta^{*}|=\frac{|G^{*}|}{2\pi f}$ we get

(1) $$\gamma_{1,13}=\frac{3\;I_{1f}h}{2\pi^{3}f^{2}D^{3}\Delta \phi \;\left|\eta^{*}\right|}\;$$

**FIGURE 7:**
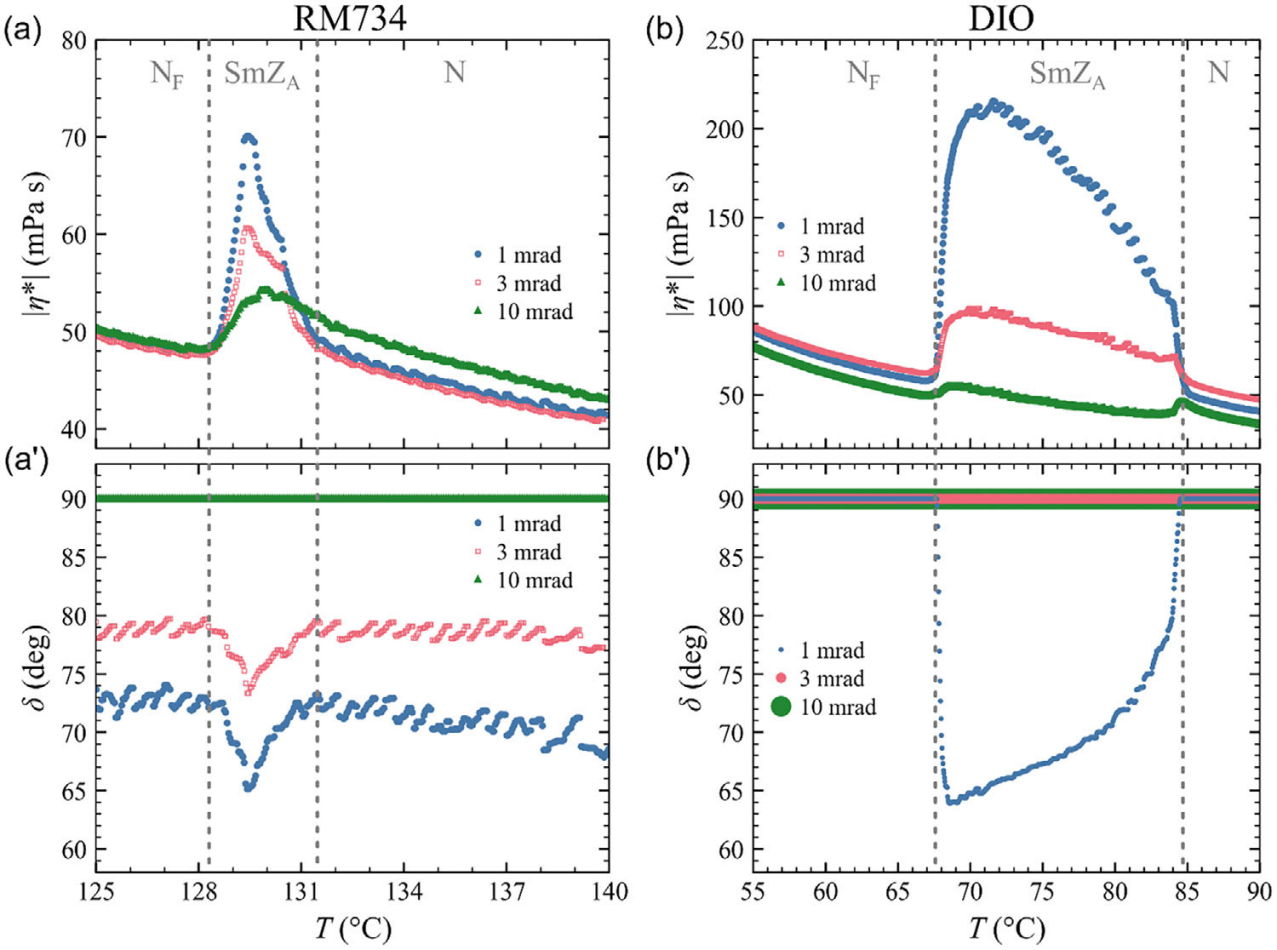
Temperature dependences of the absolute value of the complex viscosity of RM734 (a) and DIO (b) measured at ***f*** = 10 *** Hz***. The corresponding ***δ*** phase calculated from the ratio of the storage and loss modulus using ***G′′***/***G′*** = ***tan*** ***δ***.

To determine γ_1,13_, we measured the temperature dependences of the complex viscosities at *f* = 10 Hz, which are shown in Figure 7a and Figure 7b for RM734 and DIO, respectively.

In oscillatory rheometry, the viscoelastic response of a material is characterized by the complex shear modulus G*, which is related to the complex viscosity as $\eta^{*}=\frac{G^{*}}{i2\pi f}$. The real part of G* corresponds to the elastic response where the stress component is in‐phase with the strain, while the imaginary part is related to the purely viscous response characterized by the out‐of‐phase component of the stress with respect to the strain wave. In Figure 7a’ and Figure 7b’, the temperature and oscillation amplitude dependence of the angle δ is displayed, which was calculated from the viscoelastic ratio (or loss tangent, $\tan\delta =G^{\prime \prime }/G^{\prime }$) for RM734 and DIO, respectively. RM734 (see Figure 7a’) is characterized by a dominantly viscous response with δ > 70° that approaches 90° when Δϕ reaches 10 mrad. The observed decrease of δ in the SmZ_A_ phase is a sign of increasing elastic response. A similar increased elasticity was found in the SmZ_A_ phase of DIO, but only at low oscillation amplitude. This is due to the smectic‐like structure that responds elastically at low strain amplitudes but gets destroyed at higher strain amplitudes leading to purely viscous response. In DIO (Figure 7b’), purely viscous response with δ ≈ 90° was measured both in the N and N_F_ phases. Note that the δ data of RM734 is noisier (precision is lower) than of DIO due to the lower viscosity.

We also note that similar to viscoelasticity, the piezoelectricity may also be treated by complex formalism. However, its interpretation is not so straightforward as of complex shear modulus *G*, where elastic and dissipative contributions are clearly identified by the real and imaginary parts. In general, the phase of the piezoelectric response is not the same as that of *G* (compare the phases in Figure 2 and Figure 7). In the *N_F_* phase of both materials, the rheological responses are mostly viscous, i.e., $G^{\prime }\ll G^{\prime \prime }$, and δ ≈ 90°, while the first harmonic current signals have different phases depending on the material and experimental parameters such as temperature, frequency and amplitude. Although the ratio $G^{\prime \prime }/G^{\prime }$ may play a role in the phase shift of the piezoelectric current response, it is likely also influenced by the dynamics of director reorientation induced by the shear flow. The dynamics of flow alignment can be calculated by the Ericksen‐Leslie theory, which was done for non‐polar nematic liquid crystals [38, 39]. For ferroelectric nematic liquid crystals, the analysis of shear alignment is more difficult, since the electrostatic effects due to the presence of spontaneous polarization must also be considered. As a result, the phase of the shear induced current could be influenced by the viscosity, the spontaneous polarization, frequency, and other experimental conditions. The different current signals measured in RM734 and DIO (see Figure 2) may be explained by the significantly higher viscosity of DIO.

To calculate a lower limit of the piezoelectric coupling coefficient γ_1,13_ from Equation (1), we use the complex viscosity values. For RM734 at 128 ^○^C in the N_F_ phase where *I*_1*f*_ =  10 nA and η = 48 mPa · s for *f* = 10 Hz, Δϕ=1mrad with *h* = 80 µm and *D* = 25 mm results in γ_1,13_ ≈ 0.5 µC/N. For DIO at 68°C *I*_1*f*_ ≈ 20 nA and η ≈ 60 mPa · s for *f* = 10 Hz, Δϕ=1mrad, which gives γ_1,13_ ≈ 0.8 µC/N. These numbers are 1 and 2 orders of magnitudes larger than the values of γ_1,13_ obtained by converse piezoelectric measurements on a room‐temperature ferroelectric nematic liquid crystal KPA‐02 [16] at 100 Hz and 1 kHz respectively. The different values of the piezoelectric coupling constants obtained from converse piezoelectric measurements at > 100 Hz and from direct measurements below 10 Hz may be explained by factoring in the $\frac{1}{f^{2}}$ frequency dependence of Equation (1).

The experimental conditions and the proposed physical mechanism leading to γ_1,13_ are shown in Figure 1a. We propose that the physical mechanism leading to the periodic shear‐induced electric current is the flow alignment that rotates the polarization toward the electrodes leading to a periodic electric field and a periodic electric current (see Figure 1a). The measurable current is the temporal derivative of the charge difference between the top and bottom plates, which can originate from the time dependence of the polarization component along direction 1 at the electrodes, which we denote by Δ*P*(*r*, *t*) assuming radial dependence. The amplitude of the first harmonic current response is calculated as $I_{1f}=4\pi f\;\int_{o}^{D/2}\int_{0}^{2\pi }\Delta P\left(r,t\right)rdrd\phi =fD^{2}\pi^{2}\left\langle \Delta P\right\rangle$, where we estimate 〈Δ*P*〉 as an average of Δ*P*. By considering an unrealistic extreme case, when in each half of the oscillation period the spontaneous polarization is entirely oriented along direction 1, the maximum current *I*_1*f* − *max*_(*f*) would correspond to 〈Δ*P*〉_*max*_ =  *P*. This means *I*_1*f* − *max*_ (*f*) =  *fD* ^2^π^2^ *P* ≈ *f* · 6.25 · 10^−4^ π^2^ · 5 · 10^−2^ ≈ *f* · 3 · 10^−4 ^A, so for *f* = 10 Hz shear, the theoretically achievable maximum induced current is 3 mA. A more realistic assumption is to consider the ferroelectric nematic as a shear aligning material as it was inferred from previous rheological measurements [29]. Other studies indicated that the shear aligning nature of N_F_ materials is restricted to shear rates lower than about 40 s^−1^ and at higher shear rates the director aligns along the vorticity direction called the log‐rolling regime [40]. In our experiments, the shear rate was significantly below 40 s^−1^, so we stayed in the flow aligning regime. Typically, in conventional shear aligning nematic liquid crystals, the shear alignment angle of the director with respect to the bounding plane is Δθ ≈ 5°‐15°. Taking Δθ ∼6° for our N_F_ materials, 〈Δ*P*〉_*max*_ =  *P*sin 6°, providing *I*_1*f* − *max*_(10 Hz) ≈ 0.3 mA. In our experiments, the highest *I*_1*f*_ at *f* = 10 Hz was 90 nA for DIO at 68°C upon the transition to the N_F_ phase. This is 3–4 orders of magnitude smaller than the theoretical limit. Assuming completely uniform alignment and rotation of the polarization, this can be interpreted as the induced Δ*P*/*P* ≈ 3 · 10^−5^ or the flow alignment angle $\Delta \theta \approx 3\cdot 10^{-5\;}\mathrm{rad}∼0.002$ degrees. However, the induced current can be low even for much larger director rotation if the director of the polarization were random or even for uniform optical axis by assuming almost equal probability of the polarization pointing left or right. The observation that the double frequency current *I*_2*f* _overcomes the first harmonic signal *I*_1*f* _at Δϕ>4mrad, suggests that Δθ ≫ 0.1 mrad and simply the oppositely oriented polarizations cancel each other.

To obtain more information about the possible structural changes in the periodically sheared fluid, we recorded the texture of the liquid crystal as a function of the phase, α = 2 π*ft*, of the strain as seen in Video S1 for RM734 at 125°C with *f* = 10 Hz and Δ ϕ = 1 mrad. In the studied frequency and strain ranges, no significant intensity change could be detected as a function of α. We can estimate the resulting intensity modulation by assuming flow alignment discussed above. Considering the sheared fluid between crossed polarizers at 45° with respect to the flow velocity, the ξ transmittance (0 < ξ < 100%) can be calculated as ξ_Δθ_ = sin ^2^(Δψ(Δθ)/2) , where the Δψ optical phase difference is related to the Δθ tilt angle due to flow alignment assuming homogeneous tilt: $\Delta \psi \;\left(\Delta \theta \right)=\frac{2\pi }{\lambda }\;h\left(n_{e}/\sqrt{1+\frac{n_{e}^{2}-n_{o}^{2}}{n_{o}^{2}}\sin^{2}\left(\Delta \theta \right)}-n_{o}\right)$, where *n_e_* and *n_o_* are the extraordinary and ordinary refractive index, respectively. Considering *n_e_* =  1.6, *n_o_* =  1.4, λ = 660 nm and *h* = 80 µm, the planar initial state leads to Δψ(0) ≈ 48.5π. As being between a minimum (Δψ = 48π) and a maximum (Δψ = 49π) intensity range, the transmitted light intensity near the planar initial state is sensitive to the modulation of the tilt angle. In such state, the Δθ  = 3 · 10^−5 ^rad calculated above results in ξ_Δθ_ − ξ_0_ ≈ 8 · 10^−6^%, which can be difficult to detect. Even a hundred times larger tilt angle Δθ  = 3 · 10^−3 ^rad would result in hardly measurable intensity modulation: ξ_Δθ_ − ξ_0_ ≈ 8 · 10^−2^%.

It is however seen that due to the alignment layers, an inhomogeneous alignment occurs with domains of orientation nucleated at the surfaces shown by defect lines. Apparently, the effect of shearing does not result in significant changes in the orientation. Only small alterations may happen, which cannot be resolved by the applied optical detection method, however, this agrees with the above calculation referring to a low level of shear alignment.

**FIGURE 8:**
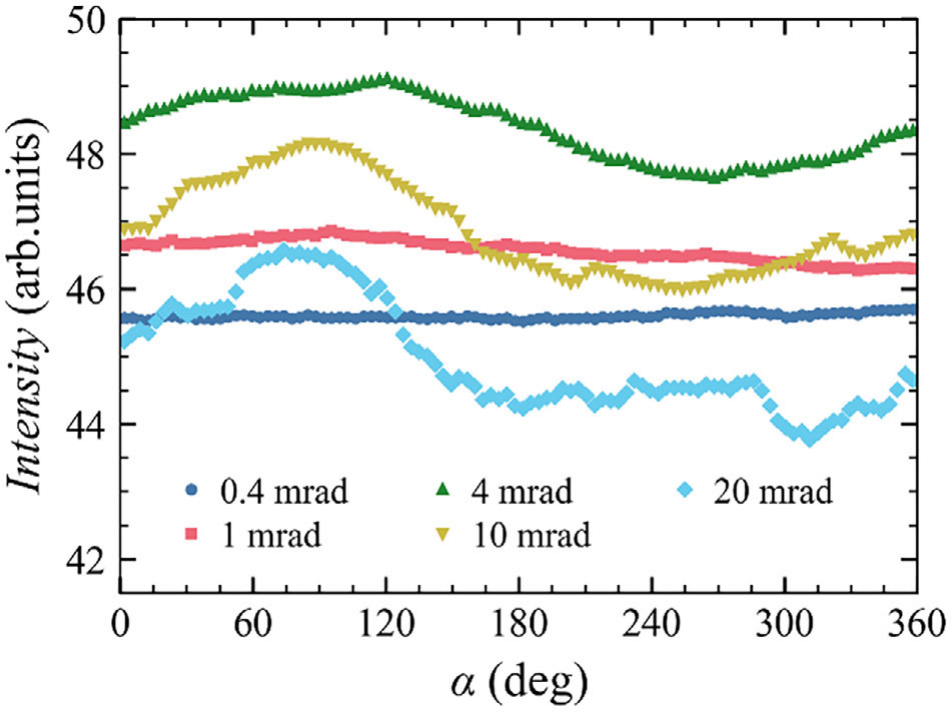
Transmitted intensity measured in RM734 at 125°C and f = 10 Hz between crossed polarizers as a function of the phase ***α*** of the oscillatory shear for different Δϕ oscillation amplitudes.

In Figure 8, we have plotted the intensity (integrated on the entire field of view) as a function of phase, α, for different Δϕ values for RM734 at 125°C and *f* = 10 Hz. The data show undetectable intensity changes at Δϕ<1mrad, while at Δϕ≥4mrad, a small (∼1%) modulation is seen. We also note that the appearance of the second harmonic signal is seen at Δϕ=20mrad. This angle is larger than what we observed from the electromechanical measurements, which indicates that the mechanical effect of the second harmonic component of the director is much stronger than its optical effect. It is because the opposite direction of the rotation adds up in the *I*_2*f*_ but tends to cancel out in the optical signal as seen in Video S2, for Δϕ=20mrad.

Surprisingly, our observations show highly augmented *I*_1*f* _signals of DIO at the N‐SmZ_A_ and SmZ_A_‐N_F_ transitions. These indicate much larger coupling between the flow and director rotation (enhanced flow alignment) at these transitions. As the SmZ_A_‐N_F_ transition is approaching, the periodicity of the smectic layers with opposite polarization directions in subsequent layers increases critically [23]. This softens the director fluctuations leading to the enhanced flow alignment. The enhancement at the N‐SmZ_A_ also suggests that the N phase already has N_F_‐like aggregations that critically increase near the transition. The existence of such domains may also explain why, although the measured current is small, the values are out of the measurement error (see Figure 2) in the N phase.

## 5 Conclusions

In this work, we have carried out periodic shear‐induced electric current measurements on the two archetypic ferroelectric nematic compounds, RM734 and DIO. From temperature, frequency and shear angle dependent results of the first and second harmonic current signals together with temperature and oscillatory rheology measurements, we were able to deduce the effective piezoelectric coupling constants. These values are similar to both materials and were compared to results of previous converse piezoelectric measurements. We also proposed a physical mechanism related to flow alignment of the ferroelectric polarization leading to the direct piezoelectric signals. Our results indicate that about two orders of magnitude increase of the shear‐induced electric current can be achieved if the ferroelectric polarization could be aligned uniformly in macroscopic (>  1 cm) size ranges. Therefore, the piezoelectric coefficients determined here are only lower estimates of an intrinsic material property. Future theoretical work by extending the Ericksen‐Leslie theory to ferroelectric nematics with electrostatic contributions may give rise to a more realistic estimate of the piezoelectric coefficient in terms of the spontaneous polarization and the viscosity coefficients.

## Author Contributions

**Péter Salamon**: conceptualization, funding acquisition, writing – review and editing, methodology, validation, visualization, software, resources, data curation, investigation. **Marcell Tibor Máthé**: methodology, software, data curation, investigation, writing – review and editing. **Hiroya Nishikawa**: resources, investigation, formal analysis. **Fumito Araoka**: resources, validation, conceptualization, funding acquisition, writing – review and editing. **Antal Jákli**: conceptualization, supervision, writing – original draft, funding acquisition, formal analysis, project administration, validation.

## Conflicts of Interest

The authors declare no conflicts of interest.

## Supporting information




**Supporting File 1**: advs77907‐sup‐0001‐VideoS1.mp4.

**Supporting File 2**: advs77907‐sup‐0002‐VideoS2.mp4.

## Data Availability

The data that support the findings of this study are available from the corresponding author upon reasonable request.
